# Design and improvement of artificial redox modules by molecular fusion of flavodoxin and flavodoxin reductase from *Escherichia coli*

**DOI:** 10.1038/srep12158

**Published:** 2015-07-16

**Authors:** Patrick J. Bakkes, Stefan Biemann, Ansgar Bokel, Marc Eickholt, Marco Girhard, Vlada B. Urlacher

**Affiliations:** 1 Institute of Biochemistry, Heinrich-Heine University Düsseldorf, Universitätsstr. 1, 40225 Düsseldorf, Germany

## Abstract

A variety of fusion proteins between the versatile redox partners flavodoxin (FldA) and flavodoxin reductase (Fpr) from *Escherichia coli* was constructed with the aim to improve the electron transfer properties. The order in which FldA and Fpr were fused and the linker region between them was varied in a systematic manner. A simple molecular tool, designated “DuaLinX”, was developed that facilitated the parallel introduction of flexible glycine-rich and rigid proline-rich linkers between the fusion partners in a single cloning event. The fusion constructs were tested for their ability to transfer electrons to cytochrome *c* and cytochrome P450 109B1 from *Bacillus subtilis*. With CYP109B1, the performance of the constructs showed, independent of the domain order, a strong dependency on linker length, whereas with cytochrome *c* this phenomenon was less pronounced. Constructs carrying linkers of ≥15 residues effectively supported the CYP109B1-catalysed hydroxylation of myristic acid. Constructs carrying proline-rich linkers generally outperformed their glycine-rich counterparts. The best construct, FldA-Fpr carrying linker ([E/L]PPPP)_4_, supported CYP109B1 activity equally well as equivalent amounts of the non-fused redox partners, while cytochrome *c* reductase activity was ~2.7-fold improved. Thus, to functionally connect redox partners, rigid proline-rich linkers may be attractive alternatives to the commonly used flexible glycine-rich linkers.

In *Escherichia coli*, the NADPH-dependent flavin adenine dinucleotide (FAD) containing flavodoxin/ferredoxin reductase, Fpr (Fig. 1b) and its dedicated redox partner flavin mononucleotide (FMN) containing flavodoxin, FldA (Fig. 1a) are required for the activation of key enzymes in the synthesis of methionine^1^, biotin^2^, pyruvate^3^ and deoxyribonucleotides^4^,^5^. Remarkably, the Fpr-FldA redox system has been shown in addition to be able to reduce a variety of non-physiological electron acceptors, which include cytochrome *c*^6^ and a number of cytochrome P450 monooxygenases (P450 or CYP). Although P450s have not been identified in *E. coli* to date^7^, Fpr-FldA can serve as a surrogate redox system for bovine CYP17A1^8^,^9^, human CYP1A2 and CYP3A4^10^,^11^, the isolated heme domain of CYP102A1 from *Bacillus megaterium* (P450 BM3)^6^ and CYP109B1 from *Bacillus subtilis*^12^.

The fact that Fpr and FldA can be highly expressed in a soluble and stable form in *E. coli*^6^,^9^,^13^ and that these enzymes can functionally interact with P450s, which are seminal targets of biotechnology^14^, makes them attractive candidates for protein engineering and biotechnological utilization. P450 enzymes are of biotechnological importance as they are able to catalyse the selective introduction of an oxygen atom from dioxygen into a wide variety of organic molecules^14^,^15^,^16^. The requirement for redox partner(s) has remained one of the challenging limitations in utilizing the full biosynthetic potential of P450 monooxygenases^14^. To tackle this problem, artificial fusion proteins are frequently constructed with the aim to reduce the multi-component nature of P450 systems and to improve the electron transfer properties^17^,^18^,^19^. Such fusion constructs are often tailored after naturally occurring P450s with covalently attached redox partners, most of which are soluble enzymes^20^ that appear to have superior catalytic activities, with P450 BM3 as best served example^21^. P450 BM3 is a catalytic self-sufficient enzyme in which the heme domain is connected to the CPR-like reductase domain via a short peptide linker. P450 BM3 combines an unprecedented high monooxygenase activity with highly efficient electron transfer as evidenced by a *k*_cat_ of 17,100 min^−1^ for the substrate arachidonic acid^22^,^23^.

Artificial fusions of redox partners and P450s are usually accomplished by introducing linker peptides between the individual components^18^,^19^,^23^. It has been generally recognized that a proper design of the linker is vital to the overall function of the fusion protein^24^. In many cases, the inherent properties of linkers occurring in natural multi-domain proteins^25^,^26^ have served as a basic guide to linker design^24^. For the construction of “artificial” fusion proteins two main types of linkers are often used: (i) flexible linkers, which mostly contain glycine and/or polar residues such as serine or threonine, and (ii) rigid linkers that are either of helical nature or enriched in proline residues^24^. Indeed, numerous studies have demonstrated that the linker length as well as linker composition are important for P450 fusion enzyme activity^17^,^18^,^19^, whereas in some cases the domain order proved to be more critical than the linker contribution^27^. It is of note that in many cases, intuitively, short flexible linkers were chosen to connect the various redox partners^27^,^28^,^29^,^30^,^31^.

Here, we report the construction of a set of “artificial” fusion proteins consisting of the physiological redox partners Fpr and FldA from *E. coli.* To study the effects on electron transfer, a variety of fusion constructs was generated in which the order of the fusion partners, the linker length and the linker type were varied in a systematic fashion in an attempt to construct a single protein with improved electron transferring properties. To facilitate linker insertion between FldA and Fpr we developed a novel molecular tool, termed “DuaLinX”, which allowed the parallel introduction of two distinct types of linkers in a single cloning event. These so-called “DuaLinX” are double stranded DNA linkers that depending on their orientation after insertion, code for either flexible (GGGGS)_n_ or rigid ([E/L]PPPP)_n_ linkers. The various fusion constructs were expressed in *E. coli*, purified, and tested *in vitro* for their ability to transfer electrons to cytochrome *c* and CYP109B1.

## Results

### Construction of molecular fusions between FldA and Fpr and implementation of DuaLinX

**Figure 1:**
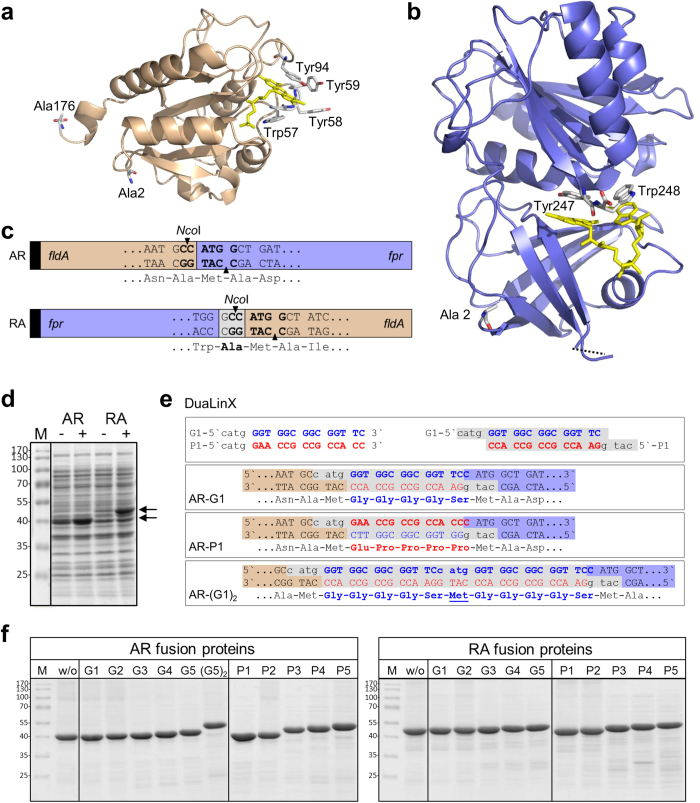
Generation of fusion constructs consisting of flavodoxin (FldA) and flavodoxin reductase (Fpr) from *E. coli.* (**a**) Ribbon representation of *E. coli* FldA (PDB 1AG9), with indicated N- (Ala2) and C-terminal (Ala176) residue. The aromatic residues surrounding the FMN cofactor (yellow) are shown as sticks. (**b**) Ribbon representation of *E. coli* Fpr (PDB 1FDR), with indicated N- (Ala2) and C-terminal (Trp248) residue. Tyr247 and Trp248 contribute to the binding of the cofactor FAD (yellow). (**c**) Representation of the genetic fusions constructed from *E. coli fldA* and *fpr* in two possible arrangements: AR (top) and RA (bottom), both containing an N-terminal His_6-_tag (black box) and a unique *Nco*I restriction that facilitates linker insertion between the fusion partners. (**d**) SDS-PAGE analysis of total cell extracts of *E. coli* BL21(DE3) cells harbouring either pET11a-*hAR* or pET11a-*hRA*, before (−) and 3 h after induction with IPTG (+); M, marker proteins with indicated molecular weight (kDa). (**e**) Schematic representation of the DuaLinX procedure. DuaLinX are generated from complementary oligonucleotide pairs that form double stranded DNA linkers with *Nco*I-compatible overhangs (top panel and Table 1). DuaLinX of different lengths (5, 10, 15, 20 and 25 residues) were created and designed such that depending on the orientation of the DuaLinX after insertion, either glycine-rich (GGGGS)_n_ or proline-rich ([E/L]PPPP)_n_ linkers are generated (n = 1–5, respectively). As an example, the insertion of the G1/P1 (n = 1) linker in the AR construct is shown. (**f**) SDS-PAGE analysis of the fusion constructs purified by IMAC. Fusion protein (2 μg) was loaded in each lane; w/o, without linker; G, (GGGGS)_n_ linker and P, ([E/L]PPPP)_n_ with numbers (n = 1–5) indicating the number of linker segments; (G5)_2_ indicates an AR fusion construct with two G5 linkers inserted in tandem.

The versatile redox partners Fpr and FldA from *E. coli* were engineered in a systematic manner through molecular fusion and a novel developed molecular tool that facilitated linker engineering, with the aim to construct a single redox module facilitating electron transfer. Several studies on fused redox enzymes have revealed that the order in which the fusion partners are connected as well as the linker region between them, can affect the overall activity of the fusion protein^28^,^29^,^30^,^32^. For this reason, the genes encoding FldA and Fpr were fused directly through overlap PCR in two possible arrangements: *fldA:fpr* (AR) and *fpr:fldA* (RA) (Fig. 1c). To facilitate linker insertion between the respective fusion partners a unique *Nco*I restriction site was introduced at the gene fusion site (Fig. 1c). Here, in principle any desired double stranded DNA fragment can be inserted as long as it contains *Nco*I-compatible overhangs. The fusion genes were cloned into pET-11a to facilitate their expression in *E. coli* and contained nucleotides encoding an N-terminal His_6_-tag to allow easy purification of the corresponding fusion proteins.

Since scientists have successfully employed both flexible- and rigid-type linkers to functionally connect a large variety of fusion partners, we decided to introduce both these types of linkers in our fusion constructs; i.e. we tested the frequently used flexible (GGGGS)_n_ linkers as well as rigid proline-rich linkers^24^. For this purpose, the palindromic nature of the *Nco*I recognition sequence and the complementarity of the codon triplets for glycine (GGU, GGC, GGA and GGG) and proline (CCA, CCG, CCU and CCC) were exploited to generate the fusion constructs with the desired linkers.

Complementary oligonucleotide pairs with *Nco*I-compatible overhangs were designed such that upon hybridization, the double stranded DNA linkers code for either a flexible glycine-rich or a rigid proline-rich linker depending on the orientation of the linker after insertion (Table 1 and Fig. 1e). These double stranded genetic elements, termed “DuaLinX”, were specifically designed to encode flexible (GGGGS)_n_ and rigid ([E/L]PPPP)_n_ linkers. To investigate the influence of the linker length on fusion protein activity DuaLinX of different lengths (n = 1–5) were created that code for linkers consisting of 5, 10, 15, 20 and 25 amino acids, respectively. For simplicity the corresponding flexible glycine-rich linkers are termed G1–G5 and the rigid proline-rich linkers are termed P1–P5, respectively (Table 1).

The cloning procedure described above in principle also allows the insertion of multiple linkers in tandem, which results in an additional methionine between the linker segments. Note that such internal methionine is absent in the original DuaLinX (Table 1). For example, the AR-(G1)_2_ fusion construct contains a double insertion of the (GGGGS)_1_ linker, which results in an extended linker with amino acid sequence GGGGS-M-GGGGS (Fig. 1e), and thus differs from the original G2 linker (GGGGS-GGGGS).

To evaluate the insertion of the DuaLinX between the redox partners, the obtained fusion constructs were analysed for the orientation and the number of linkers after insertion (Table 2). Of the 71 confirmed clones, 56% carried a glycine-rich linker and 44% carried a proline-rich linker. This small preference for linker insertion in the glycine orientation was observed for both the AR and RA fusion construct (Table 2). Importantly, when using a 1:3 molar ratio of vector to insert, the vast majority of clones (75%) contained a single linker insert, whereas 21% of the clones carried two linkers in tandem and only few clones were identified that contained a triple insert (4%). For example, the AR-(G5)_2_ fusion construct was obtained, which carries a double insertion of linker G5, thus generating a glycine-rich linker composed of 51 amino acids (Table 1). To demonstrate proof of principle, AR-(G5)_2_ was expressed, purified and tested for activity, along with the other constructs (see below).

Since insertion of multiple (up to three) DuaLinX had occurred under the tested conditions, it is expected that insertion would also occur in “mixed orientation”. These latter constructs could however not be identified. On the other hand, verification of the DNA sequence of several obtained constructs revealed a premature stop of the sequencing reaction that occurred at the linker insertion site. We hypothesize that these constructs carry multiple DuaLinX in mixed orientation. Due to the mixed orientation of the linkers large palindromic DNA sequences are formed, which likely form secondary structures that interfere with DNA polymerase function. Therefore these constructs were not further pursued.

### Expression and purification of the fusion constructs

Upon induction with IPTG, the AR and RA fusion protein without linker were produced successfully in similar amounts (Fig. 1d). After cell lysis the majority of AR was found in the soluble protein fraction, while RA was predominantly found in the insoluble protein fraction (Supplementary Fig. S1). The AR and RA constructs carrying the different linkers were expressed at levels similar to those of the parental linker-less constructs and were all present predominantly in soluble form (Supplementary Fig. S1). Thus, in case of the RA construct, the introduction of a linker between the respective redox partners resulted in a substantial improvement of protein solubility (Supplementary Fig. S1), likely caused by improved protein folding. Finally, the fusion constructs were purified from the cytosol by immobilized metal ion affinity chromatography (IMAC). SDS-PAGE analysis demonstrated that the various fusion proteins were purified to near homogeneity (Fig. 1f).

We noticed a difference in migration behaviour between the AR and RA fusion constructs during SDS-PAGE analysis (Fig. 1d,f). Compared to the AR fusions, the RA constructs contain an extra alanine at the fusion site, which results from cloning of the *Nco*I restriction site (Fig. 1c), but this obviously cannot account for the observed difference in apparent molecular weight of ~6 kDa (Fig. 1d,f). DNA sequencing confirmed however that all fusion constructs were correct. Moreover, all constructs were purified via their N-terminal His_6_-tag without appreciable protein degradation (Fig. 1f) and were shown to exhibit electron transfer activity (see below). Notably, electrospray ionisation mass spectrometry (ESI-MS) analysis of selected fusion constructs confirmed the expected theoretical masses (Supplementary Fig. S2). We therefore attribute the observed difference in migration behaviour during SDS-PAGE to an intrinsic difference at the structural level caused by the different order in which FldA and Fpr are fused together.

### Cytochrome *c* reductase activity of the fusion constructs

**Figure 2:**
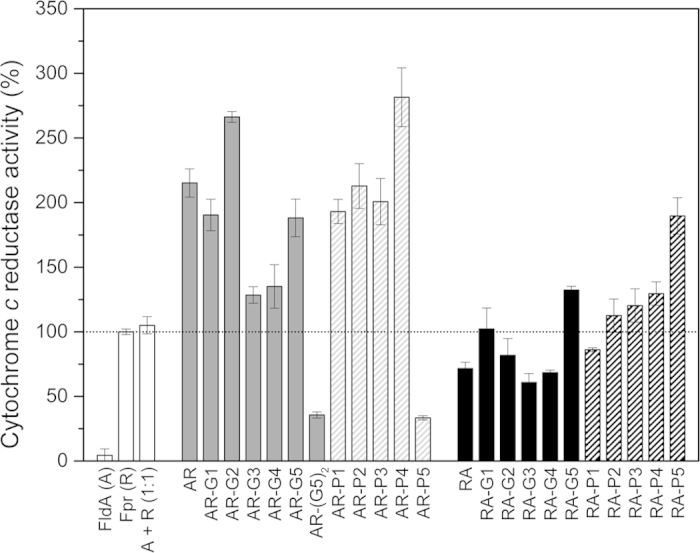
Cytochrome *c* reductase activity of fusion proteins consisting of *E. coli* FldA and Fpr. Cytochrome *c* reductase activity was determined and the data presented are the average of two independent experiments, each carried out in triplicate, with indicated standard deviation. The activity of the fusion proteins was related to the activity of the non-fused Fpr (R), which was set to 100%. The activities of the AR and RA fusion proteins are shown in grey and black, respectively, with filled bars representing constructs harbouring glycine-rich linkers and dashed bars representing constructs harbouring proline-rich linkers.

It has previously been demonstrated that Fpr exhibits NADPH-dependent reductase activity towards cytochrome *c*, and that FldA can serve as a shuttle that delivers electrons from Fpr to cytochrome *c*, thereby enhancing cytochrome *c* reduction^6^. We therefore tested the different fusion constructs for their ability to reduce cytochrome *c* (Fig. 2 and Table 3). In control reactions purified Fpr (R) catalysed the reduction of cytochrome *c* with a turnover number (*k*_*cat*_) of 42.2 ± 0.9 min^–1^, whereas FldA (A), which cannot accept electrons from NADPH directly, was incapable of reducing cytochrome *c*, as expected (Table 3 and Fig. 2). The cytochrome *c* reductase activity of the fusion proteins was related to the activity of non-fused Fpr, which was set to 100% (Fig. 2). Whereas the *k*_*cat*_ for Fpr incubated with stoichiometric amounts of FldA (enzyme ratio 1:1) was 44 min^−1^, the AR fusion protein without linker exhibited a ~2-fold higher activity, with a *k*_*cat*_ of 91 min^−1^. Thus in this case, the mere fusion of FldA to Fpr led to a substantial improvement of the cytochrome *c* reductase properties. In contrast, the RA construct, which harbours the redox partners in the reversed order, exhibited a *k*_*cat*_ of 30 min^−1^, which is lower than that of the non-fused redox partners at a 1:1 stoichiometry.

For the majority of the fusion constructs carrying a linker, the cytochrome *c* reductase activity remained about equal to that of the parent construct without linker or showed an increased activity (Fig. 2). Noticeable exceptions to this are AR-(G5)_2_ and AR-P5 (with the longest linkers), which exhibited activities that were even lower than that of the non-fused Fpr (Fig. 2). Overall, the AR fusion constructs outperformed their RA fusion equivalents (Fig. 2). In case of the RA constructs, the introduction of a linker had a general positive effect on the cytochrome *c* reductase activity. RA constructs carrying the long G5 or P5 linker, exhibited high turnover numbers of 56 min^−1^ and 80 min^−1^, respectively, corresponding to a ~1.8- and ~2.6-fold improvement over the parental linker-less construct (Table 3). Intriguingly, regardless whether a linker was present or not, the cytochrome *c* reductase activity of most of the AR fusion constructs was intrinsically high, typically exceeding that of reactions carried out with the non-fused redox partners at a 1:1 stoichiometry (Fig. 2). Notably, AR-P4 exhibited the highest activity of all fusion constructs tested, with a *k*_*cat*_ of 119 min^−1^ (Table 3 and Fig. 2). Thus, as compared to the parental AR construct without linker, the introduction of the P4 linker led to a further ~31% improvement of cytochrome *c* reductase activity, which mounts up to a ~2.7-fold improvement as compared to the non-fused redox partners (at a 1:1 stoichiometry). A similarly high cytochrome *c* reductase activity was observed for AR-G2 (*k*_*cat*_ of 112 min^−1^). Taken together the data indicate that the cytochrome *c* reductase activity of the Fpr-FldA redox system can be improved by covalent fusion of FldA and Fpr in combination with linker engineering.

### *In vitro* activity of CYP109B1 in the presence of the fusion constructs

**Figure 3:**
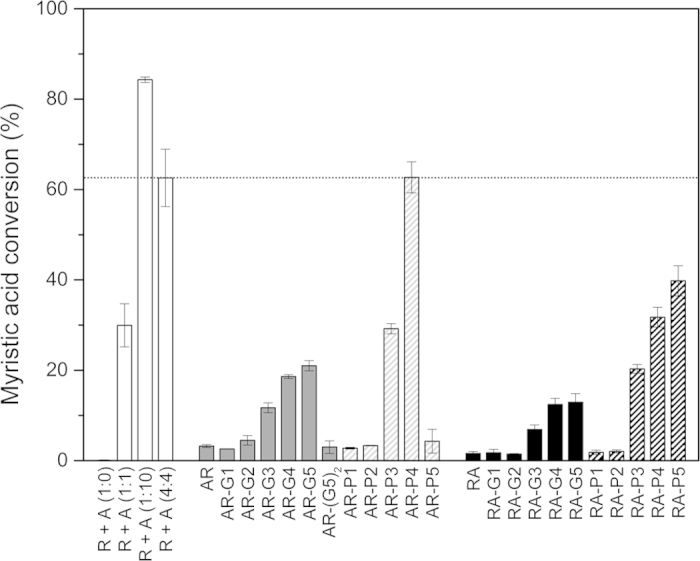
Myristic acid conversion by CYP109B1 supported by fusion constructs consisting of *E. coli* FldA and Fpr. Myristic acid conversion was determined and the data presented are average values of at least three independent conversion reactions with indicated standard deviation. Calculation of myristic acid conversion was done by GC/MS peak integration, with the total peak area of substrate plus products set to 100%. The activities of the AR and RA fusion constructs are shown in grey and black, respectively, with filled bars representing constructs harbouring glycine-rich linkers and dashed bars representing constructs harbouring proline-rich linkers.

It was previously shown that the Fpr-FldA redox system from *E. coli* is able to support the function of several P450 monooxygenases, including CYP109B1 from *B. subtilis* investigated in our group^12^. Supported by NADPH regeneration, the enzyme system consisting of Fpr, FldA and CYP109B1 at a respective stoichiometry of 1:10:1, was shown to convert ~52% of 200 μM myristic acid within 2 h^12^. To unequivocally establish that both the reductase and flavodoxin domain of the fusion constructs are functionally active, the different fusion proteins were tested for their ability to support the activity of CYP109B1 *in vitro* (Table 3 and Fig. 3). For this purpose, the fusion proteins (4 μM) were incubated in the presence of CYP109B1 (1 μM), an NADPH-regenerating system and myristic acid (200 μM) as a substrate.

In the presence of FldA and Fpr (1 μM each), CYP109B1 converted ~30% of the myristic acid in 200 min (Fig. 3). When FldA was omitted from the reaction, Fpr on its own was unable to support the conversion of myristic acid by CYP109B1 (Table 3 and Fig. 3). Thus, in contrast to the reduction of cytochrome *c* (Fig. 2), reduction of the heme-Fe of CYP109B1 requires both Fpr and FldA. When the concentration of both Fpr and FldA was increased to 4 μM, which preserves the 1:1 Fpr:FldA stoichiometry, ~63% of the myristic acid was converted, whereas in the presence of the 1:10:1 reconstituted system (10 μM FldA), a conversion of ~84% was achieved (Table 3 and Fig. 3).

Comparison of the fusion proteins to the preferred 1:10:1 reconstituted system is not straightforward as the stoichiometry of Fpr and FldA within the fusion constructs is fixed at 1:1. Consequently, the fusion constructs (4 μM) were compared to the system in which Fpr:FldA:CYP109B1 were present at a respective ratio of 4:4:1. Notably, the different fusion constructs were all able to support the conversion of myristic acid by CYP109B1, albeit with markedly different efficacies (Table 3 and Fig. 3). These results signify that both the Fpr and FldA domain of the fusion constructs are redox active.

In general, the AR fusion constructs were more effective in supporting myristic acid conversion by CYP109B1 than their RA counterparts (Fig. 3), which is consistent with the measured cytochrome *c* reductase activity (Fig. 2). The activity of the constructs without linker was however poor, supporting only 3% and 2% of myristic acid conversion in case of the AR and RA fusion construct, respectively. In contrast, AR fusion constructs carrying in particular long linkers of ≥15 residues effectively supported CYP109B1 activity (Table 3 and Fig. 3). For example ~21% and ~63% conversion of myristic acid were measured in case of AR-G5 and AR-P4, respectively. Thus, AR-P4 supported CYP109B1 activity equally well as the non-fused redox partners that were mixed at a 4:4:1 stoichiometry (Table 3 and Fig. 3). A similar dependency on the linker length was observed for the RA fusion constructs. Here, the highest activities were obtained with the constructs RA-P4 and RA-P5, supporting ~32% and ~40% myristic acid conversion, respectively. On the other hand, even though the fusion constructs AR-P5 and AR-(G5)_2_ both carry a long linker, they were unable to effectively support myristic acid conversion (Table 3, Fig. 3), which is consistent with the low cytochrome *c* reductase activity measured for these fusion proteins (Figs 2 and 3). Apparently the electron transfer ability of these fusion proteins is compromised, which might be caused by a protein folding defect.

CYP109B1 has been reported to hydroxylate fatty acids at subterminal positions yielding monohydroxylated products; in case of myristic acid the positions C11 (ω_−3_) and C12 (ω_−2_) are preferred for hydroxylation^12^. The regioselectivity of myristic acid conversion by CYP109B1 was determined for reactions carried out with the various fusion constructs (Table 3). Quantitative analysis of the detected products revealed that the carbon atoms C11 (ω_−3_) and C12 (ω_−2_) were preferred for hydroxylation regardless whether FldA and Fpr were fused to each other or not, which is in agreement with the data published previously (Table 3)^12^. Thus molecular fusion of FldA to Fpr did not affect the regioselectivity of CYP109B1.

## Discussion

The physiological redox partners Fpr and FldA from *E. coli* have been well characterized, both structurally and functionally^6^,^33^,^34^. Intriguingly, the Fpr-FldA redox system has been shown to be functionally promiscuous as it can effectively deliver electrons to non-physiological partners, which include a variety of P450 enzymes^6^,^8^,^9^,^10^,^11^. Thus Fpr-FldA may potentially serve as a universal electron transfer system, which inspired us to generate a variety of fusion proteins between FldA and Fpr in an attempt to improve the electron transfer properties.

Simple molecular tools for the construction of fusion proteins with linkers that differ in length and amino acid composition are however rather scarce. A recent report describes the P-LinK method, which is a PCR based procedure for the generation of multiple fusion constructs with variable linker lengths (1–16 amino acids) that requires only a single cloning and transformation step^35^. In order to achieve a high diversity of fusion constructs we developed a simple molecular tool that enabled the parallel introduction of two distinct types of linkers encoded by a single genetic element (Fig. 1e). These so-called DuaLinX were designed to simultaneously code for flexible (GGGGS)_n_ and rigid ([E/L]PPPP)_n_ linkers, as determined by the orientation of the DuaLinX after insertion between the fusion partners (Fig. 1e and Table 1). To assess optimal linker length DuaLinX of different lengths (n = 1–5) were created. Fine-tuning of the length of these linkers can be easily accomplished by altering the number of complementary nucleotide triplets coding for glycine/proline within the primer pairs that form the DuaLinX (Table 1). Moreover, DuaLinX can in principle be used with any proteins of interest and could therefore be combined with other established molecular tools such as “Molecular Lego”^36^,^37^, “LICRED”^38^,^39^ and “PUPPET”^40^ to further facilitate the construction of artificial fusions for P450 systems. Finally, the DuaLinX procedure is, to the best of our knowledge, the first method that enables the simultaneous generation of fusion constructs harbouring linkers of different type (flexible or rigid) and length (due to multiple linker insertion in tandem) in a single cloning event (Fig. 1).

It has previously been demonstrated that FldA, when present in excess to Fpr, can act as a single-electron shuttle that is able to stimulate the rate of Fpr-dependent cytochrome *c* reduction^6^. Interestingly, most of the AR fusion constructs exhibited a cytochrome *c* reductase activity that was substantially higher than that of the non-fused Fpr (Table 3 and Fig. 2). This stimulatory effect may indicate that the FldA domain of these fusion constructs effectively contributes to electron transfer. In a similar fashion, a fusion protein consisting of the mature forms of spinach leaf ferredoxin (N-terminal) and ferredoxin reductase (C-terminal) exhibited a high cytochrome *c* reductase activity and outperformed the non-fused redox partners^41^. In a different study, an artificial construct obtained by chemical crosslinking of FldA and Fpr exhibited reduced kinetics parameters for cytochrome *c* reduction as compared to the free redox partners^6^. We observed a similar effect when FldA was fused directly to the C-terminus of Fpr; the RA construct exhibited a ~32% lower cytochrome *c* reductase activity as compared to the non-fused redox partners (Table 3 and Fig. 2). In this respect, most AR constructs exhibited a higher cytochrome *c* reductase activity when compared to their RA equivalents, regardless whether a linker was present or not (Fig. 2). This phenomenon was also observed when CYP109B1 was used as the terminal electron acceptor. Typically, the AR fusion constructs were more effective in supporting myristic acid conversion by CYP109B1 than their RA equivalents (Fig. 3). Thus, the domain order of the fusion constructs substantially contributes to a productive supply of electrons to the terminal acceptor.

Examination of the crystal structure of Fpr (Fig. 1b) reveals that Tyr247 and Trp248 at the extreme C-terminus both play a role in the binding of the co-factor FAD^33^. Indeed, several studies on flavin reductases (EC 1.18.1.2) from different origins have revealed that replacement of the tyrosine residue that covers the isoalloxazine moiety of the cofactor by different amino acids leads to dramatic changes in nucleotide binding and electron exchange^42^,^43^,^44^. It can be envisaged that attachment of a protein at the extreme C-terminus of Fpr may impose structural restraints that could interfere with NADPH-binding and/or FAD-accessibility, which in turn would lead to a compromised reductase function of the Fpr domain. The observation that the RA fusion constructs typically exhibited lower activities than their AR equivalents (Figs 2 and 3), in which the C-terminus of Fpr is likely to be unrestraint, is consistent with this view.

However, the domain order of the fusion constructs was clearly not the only factor contributing to the effective delivery of electrons to the terminal acceptor. Whereas the AR and RA fusion construct without linker exhibited substantial cytochrome *c* reductase activity (Fig. 2), these constructs were only poorly able to support CYP109B1 activity (Fig. 3). As the reduction of Fe^3+^ to Fe^2+^ within the heme of CYP109B1 is absolutely dependent on both functional Fpr and FldA (Fig. 3), the low activity of the linker-less constructs might be attributed to impaired electron transfer from (i) the FldA domain to CYP109B1 and/or (ii) from the Fpr domain to the FldA domain. For the latter case it can be envisaged that electrons are transferred via either an inter- or intramolecular pathway. The electron transfer impairment could however be alleviated by the introduction of a suitable linker between the fusion partners. Remarkably, the CYP109B1-mediated conversion of myristic acid showed, independent of the domain order of the fusion constructs, a strong dependency on the linker length, with constructs carrying long linkers (15, 20 or 25 residues) generally outperforming constructs with short linkers (5 or 10 residues) (Fig. 3). Thus for an efficient reduction of the heme-Fe^3+^ of CYP109B1, the length of the linker between the FldA and Fpr domain was more critical than the domain order of the fusion constructs. These results are in line with the notion that electron transfer between the protein-bound prosthetic groups of the redox partners relies on the distance between the redox centres^45^. The long linkers between FldA and Fpr may provide a more optimal spatial arrangement of the respective redox centres and may facilitate the shuttling of electrons from the FldA domain to CYP109B1. However, it remains to be determined whether the FldA domain receives electrons from the attached Fpr fusion partner or from the Fpr domain of another fusion construct.

It was shown previously that the reduction of the isolated heme domain of P450 BM3 by the Fpr-FldA redox system occurred via a ping-pong mechanism rather than by formation of a ternary complex^6^. In view of this, productive electron transfer by our fusion constructs likely requires a linker that provides sufficient conformational plasticity to allow the formation of an electron transfer complex between the FldA domain and the terminal electron acceptor CYP109B1. In this respect, flexible linkers are frequently employed to join domains/proteins that require a certain degree of movement or interaction for functionality^24^, which seems especially relevant to artificial fusions of redox partners^27^,^28^,^29^,^30^,^31^. In particular flexible (GGGGS)_n_ linkers have been successfully employed to generate a variety of biologically active recombinant fusion proteins^24^,^28^,^40^. Consistent with these observations, all fusion constructs carrying (GGGGS)_n_ linkers were shown to be redox active (Figs 2 and 3). Moreover, as compared to the constructs without linker, constructs carrying (GGGGS)_n_ linkers of 15, 20 and 25 residues (n = 3–5) showed a considerable improvement in supporting the monooxygenase activity of CYP109B1 (Fig. 3). Nevertheless, in virtually all cases fusion constructs carrying proline-rich linkers outperformed their glycine-rich counterparts as functional redox partners for CYP109B1 (Fig. 3). With cytochrome *c* as terminal electron acceptor a similar phenomenon was observed, but this was less pronounced (Fig. 2). For both CYP109B1 and cytochrome *c* the highest activity was obtained with the AR-P4 construct, which carries the rigid ([E/L]PPPP)_4_ linker (Table 2 and Figs 2 and 3). Importantly, AR-P4 supported CYP109B1 activity equally well as the non-fused redox partners (at 4:4 stoichiometry), whereas cytochrome *c* reduction was improved ~2.7-fold (Fig. 3). Similarly, the introduction of long proline-rich linkers (15, 20 or 25 residues) in the RA construct, which has a less optimal domain order, also resulted in a substantial improvement of enzyme activity (Figs 2 and 3). Apparently, the proline-rich linkers were better suited to generate fusion constructs capable of effectively reducing the terminal acceptor.

Analysis of inter-domain linkers from a large variety of fusion proteins that occur in nature has indicated a high incidence of proline residues^25^,^26^,^46^. It is generally believed that the high frequency of proline residues results in structural rigidity and more effective isolation of the linker from the attached protein domains^25^,^26^. Since proline lacks an amide hydrogen to donate in hydrogen bonding, this is thought to reduce the interaction between the linker and the adjacent protein domains^25^. These observations may explain why fusion constructs carrying ([E/L]PPPP)_n_ linkers outperformed their counterparts harbouring (GGGGS)_n_ linkers. However, a contribution of the glutamic acid and leucine residues of the ([E/L]PPPP)_n_ linkers cannot be excluded.

We propose that proline-rich linkers connecting redox partners may be attractive alternatives to glycine-rich linkers in accommodating the requirements for productive electron transfer. Herein, the rigid proline-rich linkers may function as a “placeholder” keeping the interaction partners at a distance suitable for electron transfer. In addition, undesired contacts between the linker and the attached redox partners are reduced as proline residues cannot form hydrogen bonds with surrounding amino acids^25^, which in turn may facilitate electron transfer.

Taken together the data indicate that Fpr and FldA, when fused together, retained their ability to transfer electrons and to functionally interact with cytochrome *c* and CYP109B1. Activity of the fusion constructs was substantially improved by linker engineering, which was greatly facilitated by the developed DuaLinX molecular tool. Our systematic approach revealed that the domain order, the linker length as well as the linker composition contributed to the overall activity of the fusion constructs. Thus, by covalent fusion and linker engineering, the multi-component nature of the Fpr-FldA redox system was successfully reduced, while maintaining (or even improving) the high electron transfer activity of the separate fusion partners, which presents an important step towards generating a universal electron transfer system.

At present the exact nature of the electron transfer pathway(s) of the different fusion constructs remains unresolved. The FldA domain may receive electrons *intra*molecularly from the covalently attached Fpr domain, or may accept electrons *inter*molecularly from the Fpr domain of another fusion protein. This will be subject to further investigation.

## Methods

### Bacterial strains and chemicals

*E. coli* DH5α (F^−^ *supE44* Δ*lacU169*(φ*80lacZ*Δ*M15*) *hsdR17 recA1 endA1 gyrA96 thi-1 relA1*) was purchased from Invitrogen (Karlsruhe, Germany) and used for molecular cloning purposes, whereas *E. coli* BL21(DE3) (F^−^ *ompT hsdS*_*B*_(r_B_^−^ m_B_^−^) *gal dcm* (DE3)) (from Novagen Darmstadt, Germany) was used for recombinant gene expression. General molecular biology manipulations and microbiological experiments were carried out by standard methods^47^. In all cases bacteria were cultured in LB medium containing 100 μg/ml ampicillin. Restriction enzymes, T4 DNA ligase, Phusion DNA polymerase, FastAP thermosensitive alkaline phosphatase and isopropyl-β-D-thiogalactopyranoside (IPTG) were obtained from Thermo Fischer Scientific (St. Leon-Rot, Germany). NADPH was from Codexis (Jülich, Germany) and glucose-6-phosphate dehydrogenase from *Saccharomyces cerevisiae* was obtained from Roche Diagnostics (Mannheim, Germany). All other chemicals were purchased from Sigma-Aldrich (Schnelldorf, Germany). Primers/oligonucleotides were from Eurofins MWG Operon (Germany).

### Construction of fusion genes

In brief, the *E. coli* gene *fldA* lacking its stop codon was directly fused to the 5’-end of *fpr*, which conveniently generates an *Nco*I restriction site right at the fusion site (Fig. 1c). The reverse construct was obtained in a similar fashion. Here, the *fpr* gene without stop codon was fused to the 5’-end of *fldA* and three extra nucleotides (GCC) were incorporated to generate the *Nco*I restriction site required for linker insertion, which results in an extra alanine residue between Fpr and FldA (Fig. 1c). Notably, the introduction of the *Nco*I recognition sequence (CCATGG) is facilitated by the presence of the start codon of the C-terminal fusion partner. The primers used to create the fusion genes are listed in Supplementary Table S1.

The genes encoding flavodoxin (*fldA*) and flavodoxin reductase (*fpr*) from *E. coli* JM109^9^ were fused by means of PCR with overlapping primers using pET11a-*fldA*^12^ and pET16b-*fpr*^12^ as corresponding DNA-templates. The used primers are listed in Supplementary Table S1. For the construction of *fldA:fpr* the respective single genes were amplified separately using primer pairs 1/2 and 3/4 (Supplementary Table S1). The obtained PCR products were gel purified and combined in a 1:1 ratio. This mixture then served as DNA-template in a second PCR reaction performed in the absence of additional primers. After 6 cycles, primers 1 and 4 were added to the reaction and PCR was carried out for an additional 30 cycles. The obtained *fldA:fpr* fusion product was then gel purified. For the construction of the inverse fusion a similar procedure was followed, using primer pairs 5/6 and 7/8 for the separate genes and primers 5 and 8 to amplify the *fpr:fldA* fusion product (Supplementary Table S1). The fusion constructs *fldA:fpr* and *fpr:fldA* were subsequently cloned into pET11a (Novagen) using the restriction enzymes *Nde*I and *Bam*HI. The sequence of the fusion genes was verified by DNA sequencing (GATC-Biotech, Konstanz, Germany). Finally, the 5’-ends of both fusion genes were modified to encode a His_6_-tag. To this end, a PCR was carried out on pET11a-*fldA:fpr* and pET11a-*fpr:fldA* with respective primer pairs 9/8 and 10/4 (Supplementary Table S1). Subsequent cloning was carried out as described above and the fusion gene sequences of the resulting constructs, pET11a-*hAR* and pET11a-*hRA*, respectively were verified.

### Linker (DuaLinX) generation

Double stranded DNA linkers were generated from complementary pairs of commercially synthesized 5’-phosphorylated oligonucleotides that contained *Nco*I-compatible overhangs (Table 1). The complementary oligonucleotides as indicated were mixed (10 μM each) in Tris-HCl, pH 7.5 (10 mM) supplemented with NaCl (50 mM) and heated for 5 min at 95 °C in a thermomixer. Hybridization of the oligonucleotides was initiated by switching off the heater and allowing the solutions to cool down to room temperature. To allow proper hybridization of the oligonucleotide pairs, the repetitive nature of the primers (g1–g5) for the (GGGGS)_n_ linkers was reduced by shuffling the triplets used for glycine (GGT and GGC) and serine (TCC, AGC and TCT), which is particularly relevant for the long linkers. As a result the complementary primers (p1–p5) for the proline-rich linkers encode linkers with amino acid sequence ([E/L]PPPP)_n_. The double stranded DNA linkers thus created (DuaLinX) were then stored at −20 °C until use.

### Linker insertion, ligation and transformation

Prior to linker insertion, the plasmids pET11a-*hAR* and pET11a-*hRA* were linearized by treatment with *Nco*I followed by dephosphorylation using FastAP according to the manufacturer’s guidelines. Linearized plasmid was mixed with linker at a 1:3 molar ratio using a final linker concentration of 10 nM; ligation was performed in the presence of 1U T4 DNA ligase. Ligation mixture (5 μl) was mixed with *E. coli* DH5α chemi-competent cells (50 μl) for transformation. The isolated plasmids were verified for linker insertion by sequencing (GATC, Konstanz, Germany). For subsequent expression studies, the verified constructs (Supplementary Table S2) were used for transformation of chemi-competent *E. coli* BL21(DE3) cells.

### Expression and purification of redox proteins

Expression and purification of CYP109B1 was carried out as described previously^48^. Flavodoxin was expressed from pET11a-*his:fldA*, whereas flavodoxin reductase was expressed from pET11a-*his:fpr* (Supplementary Table S2). Both redox proteins contain an N-terminal His_6_-tag. Expression conditions were as follows. Cultures were inoculated at an OD_600_ of 0.05 and grown till an OD_600_ ~0.6 at 37 °C and 180 rpm. Expression was induced by the addition of IPTG to a final concentration of 0.1 mM. Expression of FldA was carried out at 25 °C for 4 h and expression of Fpr was performed at 20 °C for 6 h. For the expression of the various fusion constructs (Supplementary Table S2), cultures were inoculated at an OD_600_ of 0.08 and grown till an OD_600_ ~0.6 at 25 °C and 180 rpm. Expression was induced by the addition of IPTG to a final concentration of 0.1 mM and carried out for 3 h. In all cases cells were harvested by centrifugation at 8,000 × g for 15 min at 4 °C. The supernatant was removed and the cell pellets were suspended in 8 ml buffer containing Tris-HCl, pH 7.5 (50 mM) and PMSF (0.1 mM). Cells were then disrupted by sonication on ice (four cycles of 1 min sonication followed by a 1 min pause) and cell debris was removed by centrifugation (20,000x g for 30 min at 4 °C). The soluble protein fraction was recovered and filtered through a 0.45 μm filter and used for immobilized metal ion affinity chromatography (IMAC) purification. Purification of the various redox proteins was carried out as described in the Supplementary Information.

### Determination of protein concentration

The concentration of purified CYP109B1 was determined using the CO-difference spectral assay as described by Omura and Sato (1964) using ε_450–490_ = 91 mM^−1^cm^−1^ 49,50. The concentration of Fpr was calculated by using the published extinction coefficient ε_456_ *=* 7.100 M^−1^cm^−1^ 6, whereas for FldA, ε_465_ = 8420 M^−1^cm^−1 1^ was used. For the calculation of the fusion constructs concentration, the FldA extinction coefficient at 456 nm was determined first and found to be 8.040 M^−1^cm^−1^. The extinction coefficient of the fusion constructs was then obtained by addition of the ε_456_ of Fpr and FldA, yielding ε_456_ = 15.140 M^−1^cm^−1^ (for further explanation see Supplementary Information). Alternatively, the total protein concentration was determined by a Bradford based assay^51^ (Roti-Quant, Carl Roth, Germany), with bovine serum albumin as a standard.

### Cytochrome ***c*** reductase activity assay

Cytochrome *c* reductase activity was determined for reaction mixtures containing Tris-HCl, pH 7.5 (50 mM), cytochrome *c* (50 μM, equine heart, Sigma) and redox protein (20 nM) as indicated. Reactions were started by the addition of NADPH to a final concentration of 100 μM. The final reaction volume was 1 ml. Cytochrome *c* reductase activity was determined by measuring the increase in absorbance at 550 nm^52^. Reactions were carried out in triplicate and the data presented are the average of two independent experiments, with the activity of Fpr set to 100%. To calculate *k*_cat_, a Δε_550_ = 21,100 M^−1^ cm^−1^ was used for cytochrome *c* as provided by the manufacturer (Sigma^53^).

### Myristic acid conversion and product analysis

Reconstitution of CYP109B1 activity was carried out *in vitro* in the presence of the various redox proteins as indicated. For product identification and determination of substrate conversion, 200 μl reaction mixtures were prepared, which contained Tris-HCl, pH 7.5 (50 mM), glucose-6-phosphate (4 mM), MgCl_2_ (1 mM), glucose-6-phosphate dehydrogenase from *Saccharomyces cerevisiae* (1 U, for regeneration of NADPH), CYP109B1 (1 μM) and redox protein as indicated. Reactions were started by the addition of 44 μl of a mixture containing NADPH, myristic acid and DMSO (10% (v/v)). The final concentrations of myristic acid and NADPH both were 200 μM. Reaction mixtures were incubated for 200 min at 30 °C and 250 rpm in a thermo-shaker. Reactions were stopped by the addition of HCl (8 μl, 37% (w/w)). The reaction mixtures were extracted two times with diethyl ether (0.5 ml). The organic phases were combined and then dried with anhydrous MgSO_4_. The dried organic phases (1 ml) were evaporated and the residues were dissolved in *N,O*-bis(trimethylsilyl)trifluoroacetamide (40 μl) containing trimethylchlorosilane (1% (v/v)) for derivatization. Samples were then transferred into GC vials, and incubated at 80 °C for 30 min prior to GC/MS analysis. Myristic acid conversion was analyzed on a GC/MS-QP2010 (Shimadzu, Tokyo, Japan) equipped with a FS-Supreme-5 column (30 m × 0.25 mm × 0.25 μm, CS-Chromatographie Service GmbH, Langerwehe, Germany) as described previously^54^. Estimation of myristic acid conversion and product distribution was done by GC/MS peak integration, with the total peak area of substrate plus products set to 100%. Presented myristic acid conversion data are average values of 3–6 independent conversion reactions with indicated standard deviation.

## Additional Information

**How to cite this article**: Bakkes, P. J. *et al.* Design and improvement of artificial redox modules by molecular fusion of flavodoxin and flavodoxin reductase from *Escherichia coli*. *Sci. Rep.* **5**, 12158; doi: 10.1038/srep12158 (2015).

## Supplementary Material

Supplementary Information

## Figures and Tables

**Table 1:** DuaLinX: complementary oligonucleotide pairs forming double stranded DNA linkers encoding both glycine-rich and proline-rich amino acid sequences.

g1	*5’-CATGGGTGGCGGCGGTTC-3’*
p1	*5’-CATGGAACCGCCGCCACC-3’*
G1/P1	→ GGGGS/EPPPP (n = 1, 5 amino acids)
g2	*5’-CATGGGTGGCGGCGGTAGCGGCGGTGGTGGCTC-3’*
p2	*5’-CATGGAGCCACCACCGCCGCTACCGCCGCCACC-3’*
G2/P2	→ GGGGS-GGGGS/EPPPP-LPPPP (n = 2, 10 amino acids)
g3	*5’-CATGGGTGGCGGCGGTAGCGGTGGCGGCGGTTCTGGTGGCGGCGGTTC-3’*
p3	*5’-CATGGAACCGCCGCCACCAGAACCGCCGCCACCGCTACCGCCGCCACC-3’*
G3/P3	→ GGGGS-GGGGS-GGGGS/EPPPP-EPPPP-LPPPP (n = 3, 15 amino acids)
g4	*5’-CATGGGTGGCGGCGGTTCTGGCGGTGGTGGCAGCGGTGGCGGCGGTAGCGGCGGTGGTGGCTC-3’*
p4	*5’-CATGGAGCCACCACCGCCGCTACCGCCGCCACCGCTGCCACCACCGCCAGAACCGCCGCCACC-3’*
G4/P4	→ GGGGS-GGGGS-GGGGS-GGGGS/EPPPP-LPPPP-LPPPP-EPPPP (n = 4, 20 amino acids)
g5	*5’-CATGGGTGGCGGCGGTAGCGGTGGCGGCGGTAGCGGTGGCGGCGGTAGCGGTGGCGGCGGTTCTGGCGGTGGTGGCTC-3’*
p5	*5’-CATGGAGCCACCACCGCCAGAACCGCCGCCACCGCTACCGCCGCCACCGCTACCGCCGCCACCGCTACCGCCGCCACC-3’*
G5/P5	→ GGGGS-GGGGS-GGGGS-GGGGS-GGGGS/EPPPP-EPPPP-LPPPP-LPPPP-LPPPP (n = 5, 25 amino acids)
(G5)_2_	→ GGGGS-GGGGS-GGGGS-GGGGS-GGGGS-M-GGGGS-GGGGS-GGGGS-GGGGS-GGGGS (2 × G5 in tandem: 51 amino acids)

Depending on the orientation after insertion the DuaLinX code for either (GGGGS)n or ([E/L]PPPP)n linkers. Underlined nucleotides indicate *Nco*I-compatible overhangs.

**Table 2:** Evaluation of the number and orientation of the linkers after DuaLinX insertion.

DuaLinX orientation	AR fusion constructs		RA fusion constructs		All fusion constructs
	(GGGGS)_n_	([E/L]PPPP)_n_	(GGGGS)_n_	([E/L]PPPP)_n_	(GGGGS)_n_ and ([E/L]PPPP)_n_
Single inserts	18/36 (50%)	9/36 (25%)	14/35 (40%)	12/35 (34%)	53/71 (75%)
Double inserts	2/36 (6%)	5/36 (14%)	5/35 (14%)	3/35 (9%)	15/71 (21%)
Triple inserts	0/36 (0%)	2/36 (5%)	1/35 (3%)	0/35 (0%)	3/71 (4%)
Total inserts	20/36 (56%)	16/36 (44%)	20/35 (57%)	15/35 (43%)	71/71 (100%)

Constructs obtained after ligation of the five different DuaLinX employed in our studies ((GGGGS)_n_/([E/L]PPPP)_n_, n = 1–5) were analysed for the number and orientation of the linker inserts.

**Table 3:** Catalytic properties of FldA, Fpr and selected fusion constructs.

Redox protein(s)	Cytochrome c reductase activity k_cat_ (min^−1^)	Myristic acid conversion (%)	Myristic acid product distribution (%)				
			ω_−1_	ω_−2_	ω_−3_	ω_−4_	ω_−5_
FldA (A)	0	N.D.	N.D.	N.D.	N.D.	N.D.	N.D.
Fpr (R)	42.2 ± 0.9	0	−	−	−	−	−
Fpr + FldA (1:1)	44.3 ± 2.8	30.0 ± 4.8	16.5	29.0	28.4	18.3	7.8
Fpr + FldA (1:10)	N.D.	84.3 ± 0.6	16.5	28.3	27.9	18.2	9.2
Fpr + FldA (4:4)	N.D.	62.6 ± 7.4	16.7	28.8	28.6	18.8	7.1
AR	90.7 ± 4.6	3.2 ± 0.3	+	+	+	+	−
AR-G2	112.3 ± 1.7	4.5 ± 1.1	16.7	26.7	28.3	21.8	6.5
AR-G5	79.3 ± 6.1	21.0 ± 1.1	16.6	29.3	28.6	17.6	8.0
AR-P4	118.7 ± 9.6	62.7 ± 3.4	16.7	29.5	28.8	18.3	6.6
RA	30.2 ± 2.1	1.6 ± 0.4	+	+	+	+	−
RA-G5	55.8 ± 1.2	12.9 ± 1.9	16.2	28.3	27.6	17.5	10.5
RA-P5	80.0 ± 5.9	39.8 ± 3.3	17.1	30.2	28.8	17.6	6.3

Cytochrome *c* reductase data are average values of two independent experiments, each carried out in triplicate, with indicated standard deviation. Myristic acid conversion data represent average values of at least three independent conversion reactions with indicated standard deviation. N.D., not determined; −, compound was not detected; +, compound was detected, but quantitative analysis could not be done due to low conversion of the substrate. The standard deviation for myristic acid product distribution was in all cases ≤2.7%.

## References

[b1] Fujii K. & Huennekens F. M. Activation of methionine synthetase by a reduced triphosphopyridine nucleotide-dependent flavoprotein system. J. Biol. Chem. 249, 6745–6753 (1974). 4154078

[b2] Birch O. M., Fuhrmann M. & Shaw N. M. Biotin synthase from *Escherichia coli*, an investigation of the low molecular weight and protein components required for activity *in vitro*. J. Biol. Chem. 270, 19158–19165 (1995). 7642583 10.1074/jbc.270.32.19158

[b3] Blaschkowski H. P., Neuer G., Ludwig-Festl M. & Knappe J. Routes of flavodoxin and ferredoxin reduction in *Escherichia coli*. CoA-acylating pyruvate: flavodoxin and NADPH: flavodoxin oxidoreductases participating in the activation of pyruvate formate-lyase. Eur. J. Biochem. 123, 563–569 (1982). 7042345

[b4] Bianchi V. *et al.* Flavodoxin is required for the activation of the anaerobic ribonucleotide reductase. Biochem. Biophys. Res. Commun. 197, 792–797 (1993). 8267617 10.1006/bbrc.1993.2548

[b5] Bianchi V. *et al.* *Escherichia coli* ferredoxin NADP^+^ reductase: activation of *E. coli* anaerobic ribonucleotide reduction, cloning of the gene (*fpr*), and overexpression of the protein. J. Bacteriol. 175, 1590–1595 (1993). 8449868 10.1128/jb.175.6.1590-1595.1993 PMC203951

[b6] McIver L. *et al.* Characterisation of flavodoxin NADP^+^ oxidoreductase and flavodoxin; key components of electron transfer in *Escherichia coli*. Eur. J. Biochem. 257, 577–585 (1998). 9839946 10.1046/j.1432-1327.1998.2570577.x

[b7] Lamb D. C. *et al.* The cytochrome P450 complement (CYPome) of *Streptomyces coelicolor* A3(2). J. Biol. Chem. 277, 24000–24005 (2002). 11943767 10.1074/jbc.M111109200

[b8] Barnes H. J., Arlotto M. P. & Waterman M. R. Expression and enzymatic activity of recombinant cytochrome P450 17 alpha-hydroxylase in *Escherichia coli*. Proc. Natl Acad. Sci. USA . 88, 5597–5601 (1991). 1829523 10.1073/pnas.88.13.5597 PMC51924

[b9] Jenkins C. M. & Waterman M. R. NADPH-flavodoxin reductase and flavodoxin from *Escherichia coli*: characteristics as a soluble microsomal P450 reductase. Biochemistry 37, 6106–6113 (1998). 9558349 10.1021/bi973076p

[b10] Dong M. S., Yamazaki H., Guo Z. & Guengerich F. P. Recombinant human cytochrome P450 1A2 and an N-terminal-truncated form: construction, purification, aggregation properties, and interactions with flavodoxin, ferredoxin, and NADPH-cytochrome P450 reductase. Arch. Biochem. Biophys. 327, 11–19 (1996). 8615680 10.1006/abbi.1996.0086

[b11] Yamazaki H., Ueng Y. F., Shimada T. & Guengerich F. P. Roles of divalent metal ions in oxidations catalyzed by recombinant cytochrome P450 3A4 and replacement of NADPH-cytochrome P450 reductase with other flavoproteins, ferredoxin, and oxygen surrogates. Biochemistry 34, 8380–8389 (1995). 7599128 10.1021/bi00026a020

[b12] Girhard M., Klaus T., Khatri Y., Bernhardt R. & Urlacher V. B. Characterization of the versatile monooxygenase CYP109B1 from *Bacillus subtilis*. Appl. Microbiol. Biotechnol. 87, 595–607 (2010). 20186410 10.1007/s00253-010-2472-z

[b13] Jenkins C. M. & Waterman M. R. Flavodoxin and NADPH-flavodoxin reductase from *Escherichia coli* support bovine cytochrome P450c17 hydroxylase activities. J. Biol. Chem. 269, 27401–27408 (1994). 7961651

[b14] Urlacher V. B. & Girhard M. Cytochrome P450 monooxygenases: an update on perspectives for synthetic application. Trends Biotechnol. 30, 26–36 (2012). 21782265 10.1016/j.tibtech.2011.06.012

[b15] Lewis J. C., Coelho P. S. & Arnold F. H. Enzymatic functionalization of carbon-hydrogen bonds. Chem. Soc. Rev. 40, 2003–2021 (2011). 21079862 10.1039/c0cs00067a PMC3064445

[b16] Bernhardt R. Cytochromes P450 as versatile biocatalysts. J. Biotechnol. 124, 128–145 (2006). 16516322 10.1016/j.jbiotec.2006.01.026

[b17] McLean K. J., Girvan H. M. & Munro A. W. Cytochrome P450/redox partner fusion enzymes: biotechnological and toxicological prospects. Expert Opin. Drug Metab. Toxicol. 3, 847–863 (2007). 18028029 10.1517/17425255.3.6.847

[b18] Hlavica P. Assembly of non-natural electron transfer conduits in the cytochrome P450 system: a critical assessment and update of artificial redox constructs amenable to exploitation in biotechnological areas. Biotechnol. Adv. 27, 103–121 (2009). 18976700 10.1016/j.biotechadv.2008.10.001

[b19] Sadeghi S. J. & Gilardi G. Chimeric P450 enzymes: activity of artificial redox fusions driven by different reductases for biotechnological applications. Biotechnol. Appl. Biochem. 60, 102–110 (2013). 23586997 10.1002/bab.1086

[b20] Hannemann F., Bichet A., Ewen K. M. & Bernhardt R. Cytochrome P450 systems—biological variations of electron transport chains. Biochim. Biophys. Acta 1770, 330–344 (2007). 16978787 10.1016/j.bbagen.2006.07.017

[b21] Munro A. W., Girvan H. M. & McLean K. J. Variations on a (t)heme-novel mechanisms, redox partners and catalytic functions in the cytochrome P450 superfamily. Nat. Prod. Rep. 24, 585–609 (2007). 17534532 10.1039/b604190f

[b22] Whitehouse C. J., Bell S. G. & Wong L. L. P450(BM3) (CYP102A1): connecting the dots. Chem. Soc. Rev. 41, 1218–1260 (2012). 22008827 10.1039/c1cs15192d

[b23] Munro A. W., Girvan H. M. & McLean K. J. Cytochrome P450-redox partner fusion enzymes. Biochim. Biophys. Acta 1770, 345–359 (2007). 17023115 10.1016/j.bbagen.2006.08.018

[b24] Chen X., Zaro J. L. & Shen W. C. Fusion protein linkers: property, design and functionality. Adv. Drug. Deliv. Rev. 65, 1357–1369 (2013). 23026637 10.1016/j.addr.2012.09.039 PMC3726540

[b25] George R. A. & Heringa J. An analysis of protein domain linkers: their classification and role in protein folding. Protein Eng. 15, 871–879 (2002). 12538906 10.1093/protein/15.11.871

[b26] Argos P. An investigation of oligopeptides linking domains in protein tertiary structures and possible candidates for general gene fusion. J. Mol. Biol. 211, 943–958 (1990). 2313701 10.1016/0022-2836(90)90085-Z

[b27] Sibbesen O., De Voss J. J. & Montellano P. R. Putidaredoxin reductase-putidaredoxin-cytochrome P450cam triple fusion protein. Construction of a self-sufficient *Escherichia coli* catalytic system. J. Biol. Chem. 271, 22462–22469 (1996). 8798411 10.1074/jbc.271.37.22462

[b28] Cao P. R., Bulow H., Dumas B. & Bernhardt R. Construction and characterization of a catalytic fusion protein system: P-450(11beta)-adrenodoxin reductase-adrenodoxin. Biochim. Biophys. Acta 1476, 253–264 (2000). 10669790 10.1016/s0167-4838(99)00243-5

[b29] Mandai T., Fujiwara S. & Imaoka S. Construction and engineering of a thermostable self-sufficient cytochrome P450. Biochem. Biophys. Res. Commun. 384, 61–65 (2009). 19389383 10.1016/j.bbrc.2009.04.064

[b30] Harikrishna J. A., Black S. M., Szklarz G. D. & Miller W. L. Construction and function of fusion enzymes of the human cytochrome P450scc system. DNA Cell Biol. 12, 371–379 (1993). 8517924 10.1089/dna.1993.12.371

[b31] Wittenberg G., Sheffler W., Darchi D., Baker D. & Noy D. Accelerated electron transport from photosystem I to redox partners by covalently linked ferredoxin. Phys. Chem. Chem. Phys. 15, 19608–19614 (2013). 24129892 10.1039/c3cp53264j

[b32] Lacour T. & Ohkawa H. Engineering and biochemical characterization of the rat microsomal cytochrome P4501A1 fused to ferredoxin and ferredoxin-NADP(+) reductase from plant chloroplasts. Biochim. Biophys. Acta. 1433, 87–102 (1999). 10446362 10.1016/s0167-4838(99)00154-5

[b33] Ingelman M., Bianchi V. & Eklund H. The three-dimensional structure of flavodoxin reductase from *Escherichia coli* at 1.7 A resolution. J. Mol. Biol. 268, 147–157 (1997). 9149148 10.1006/jmbi.1997.0957

[b34] Hoover D. M. & Ludwig M. L. A flavodoxin that is required for enzyme activation: the structure of oxidized flavodoxin from *Escherichia coli* at 1.8 A resolution. Protein Sci. 6, 2525–2537 (1997). 9416602 10.1002/pro.5560061205 PMC2143625

[b35] Belsare K. D. *et al.* P-LinK: A method for generating multicomponent cytochrome P450 fusions with variable linker length. Biotechniques 57, 13–20 (2014). 25005689 10.2144/000114187

[b36] Sadeghi S. J., Meharenna Y. T., Fantuzzi A., Valetti F. & Gilardi G. Engineering artificial redox chains by molecular ‘Lego’. Faraday Discuss. 116, 135–153 (2000). 10.1039/b003180l 11197475

[b37] Gilardi G. *et al.* Molecular Lego: design of molecular assemblies of P450 enzymes for nanobiotechnology. Biosens. Bioelectron. 17, 133–145 (2002). 11742744 10.1016/s0956-5663(01)00286-x

[b38] Sabbadin F., Grogan G. & Bruce N. C. LICRED: a versatile drop-in vector for rapid generation of redox-self-sufficient cytochromes P450. Methods Mol. Biol. 987, 239–249 (2013). 23475682 10.1007/978-1-62703-321-3_20

[b39] Sabbadin F. *et al.* LICRED: a versatile drop-in vector for rapid generation of redox-self-sufficient cytochrome P450s. Chembiochem. 11, 987–994 (2010). 20425752 10.1002/cbic.201000104

[b40] Hirakawa H. & Nagamune T. Use of *Sulfolobus solfataricus* PCNA subunit proteins to direct the assembly of multimeric enzyme complexes. Methods Mol. Biol. 978, 149–163 (2013). 23423895 10.1007/978-1-62703-293-3_11

[b41] Aliverti A. & Zanetti G. A three-domain iron-sulfur flavoprotein obtained through gene fusion of ferredoxin and ferredoxin-NADP+ reductase from spinach leaves. Biochemistry 36, 14771–14777 (1997). 9398197 10.1021/bi971791t

[b42] Deng Z. *et al.* A productive NADP+ binding mode of ferredoxin-NADP + reductase revealed by protein engineering and crystallographic studies. Nat. Struct. Biol. 6, 847–853 (1999). 10467097 10.1038/12307

[b43] Piubelli L. *et al.* Competition between C-terminal tyrosine and nicotinamide modulates pyridine nucleotide affinity and specificity in plant ferredoxin-NADP(+) reductase. J. Biol. Chem. 275, 10472–10476 (2000). 10744737 10.1074/jbc.275.14.10472

[b44] Nogues I. *et al.* Role of the C-terminal tyrosine of ferredoxin-nicotinamide adenine dinucleotide phosphate reductase in the electron transfer processes with its protein partners ferredoxin and flavodoxin. Biochemistry 43, 6127–6137 (2004). 15147197 10.1021/bi049858h

[b45] Moser C. C., Keske J. M., Warncke K., Farid R. S. & Dutton P. L. Nature of biological electron transfer. Nature 355, 796–802 (1992). 1311417 10.1038/355796a0

[b46] Tanaka T., Yokoyama S. & Kuroda Y. Improvement of domain linker prediction by incorporating loop-length-dependent characteristics. Biopolymers 84, 161–168 (2006). 16134173 10.1002/bip.20361

[b47] Sambook J. & Russell D. W. Molecular cloning: a laboratory manual, 3nd ed . (Cold Spring Harbor Laboratory Press, 2001).

[b48] Girhard M. *et al.* Regioselective biooxidation of (+)-valencene by recombinant E. coli expressing CYP109B1 from Bacillus subtilis in a two-liquid-phase system. Microb. Cell Fact. 8, 36 (2009). 19591681 10.1186/1475-2859-8-36 PMC2717049

[b49] Omura T. & Sato R. The Carbon Monoxide-Binding Pigment of Liver Microsomes. I. Evidence for Its Hemoprotein Nature. J. Biol. Chem. 239, 2370–2378 (1964a). 14209971

[b50] Omura T. & Sato R. The Carbon Monoxide-Binding Pigment of Liver Microsomes. II. Solubilization, Purification, and Properties. J. Biol. Chem. 239, 2379–2385 (1964b). 14209972

[b51] Bradford M. M. A rapid and sensitive method for the quantitation of microgram quantities of protein utilizing the principle of protein-dye binding. Anal. Biochem. 72, 248–254 (1976). 942051 10.1016/0003-2697(76)90527-3

[b52] Vermilion J. L. & Coon M. J. Highly purified detergent-solubilized NADPH-cytochrome P-450 reductase from phenobarbital-induced rat liver microsomes. Biochem. Biophys. Res. Commun. 60, 1315–1322 (1974). 4153862 10.1016/0006-291x(74)90341-6

[b53] van Gelder B. & Slater E. C. The extinction coefficient of cytochrome *c*. Biochim. Biophys. Acta 58, 593–595 (1962). 13897582 10.1016/0006-3002(62)90073-2

[b54] Girhard M., Tieves F., Weber E., Smit M. S. & Urlacher V. B. Cytochrome P450 reductase from Candida apicola: versatile redox partner for bacterial P450s. Appl. Microbiol. Biotechnol. 97, 1625–1635 (2013). 22526787 10.1007/s00253-012-4026-z

