# Supplementary material for: Design and improvement of artificial redox modules by molecular fusion of flavodoxin and flavodoxin reductase from Escherichia coli
Source: Sci Rep. 2015 Jul 16;5:12158. doi: 10.1038/srep12158 (PMC4503991; doi:10.1038/srep12158)
Supplement: Supplementary Information [file srep12158-s1.pdf]

Supplementary information to

Design and improvement of artificial redox modules by molecular fusion of flavodoxin and flavodoxin reductase from *Escherichia coli*

Patrick J. Bakkes[1], Stefan Biemann[1], Ansgar Bokel[1], Marc Eickholt[1], Marco Girhard[1], and Vlada B. Urlacher[1*]

[1] Institute of Biochemistry, Heinrich-Heine University Düsseldorf, Universitätsstr. 1, 40225 Düsseldorf, Germany

[*] corresponding author: Vlada B. Urlacher (vlada.urlacher@uni-duesseldorf.de)

Supplementary Methods

Immobilized metal ion affinity chromatography (IMAC). FldA and Fpr were purified separately using two 5 ml HisTrap ff crude columns (GE Healthcare in series that were equilibrated in buffer A (50 mM Tris-HCl pH 7.5 and 500 mM NaCl). The filtered protein fraction was applied to the columns and non-specifically bound proteins were eluted with buffer A containing 30 mM imidazole. Hereafter, elution of FldA and Fpr was carried out with buffer A containing 200 mM and 150 mM imidazole, respectively. The eluted proteins were then concentrated using centrifugal devices (Sartorius, Vivaspin 10 kDa MWCO), followed by desalting by employing a Sephadex G-25 column (GE-Healthcare) using buffer B (50 mM Tris-HCl, pH 7.5, 50 mM NaCl and 5% (v/v) glycerol) as eluent. Finally, purified FldA and Fpr was concentrated as described above and stored at -20°C. For the rapid small scale purification of the fusion proteins, five self-cast Ni^{2+}-NTA (Qiagen, Hilden, Germany) columns with a 1.5 ml bed volume were used in parallel. The soluble protein fractions were incubated for 1 h on ice in the presence of FAD and FMN, each at a final concentration of 10 µM and then filtered (0.45µM). The filtered protein samples were 1:1 diluted with buffer C (100 mM Tris-HCl pH 7.5 and 300 mM NaCl) and applied to the Ni^{2+}-NTA columns. Non-specifically bound proteins were removed by using buffer C containing 10 mM imidazole, whereas subsequent elution of the fusion proteins was carried out in buffer C containing 150 mM imidazole. The obtained elution fraction (~8 ml) for each fusion protein was concentrated to a volume of ~1 ml using centrifugal devices (MWCO of 30 kDa) and then desalted by gravity flow PD-10 columns (GE-Healthcare) in buffer B. Finally, the purified fusion proteins were concentrated as described above and stored at -20°C.

Determination of the protein concentration of the fusion constructs. Considering the various fusion proteins, both the FMN-containing FldA domain and FAD-containing Fpr domain contribute to absorbance. It has previously been shown that the cumulative absorbance spectrum of Fpr and FldA measured in separate chambers of a tandem cuvette only marginally differs from the absorbance spectrum obtained after mixing the two redox proteins[1], which demonstrates that major spectral changes do not occur when Fpr and FldA are allowed to interact. Therefore, for the calculation of the fusion protein concentration, the FldA extinction coefficient at 456 nm was determined first (8.040 $M^{-1}cm^{-1}$) and added to the published ε_{456} of Fpr (7.100 $M^{-1}cm^{-1}$), which yields a combined ε_{456} of 15.140 $M^{-1}cm^{-1}$ for the fusion constructs.

Supplementary Results

Supplementary Figure S1. SDS-PAGE analysis of the expression of the various fusion constructs. For the expression of the various fusion constructs cultures were prepared and treated as described in the methods section of the main manuscript. After cell disruption lysates were centrifuged at 20,000x g for 30 min at 4°C. Both the soluble (s) and pellet (p) fraction were analysed by 12.5% SDS-PAGE. 15 µg of total protein was loaded per lane. M, marker proteins with indicated molecular weights in kDa; w/o, fusion construct without linker; G1, P1, etc. indicates fusion constructs harbouring linkers with $(GGGGS)_n$ and $([E/L]PPPP)_n$ amino acid sequence (n = 1 – 5), respectively. The AR-$(G5)_2$ construct (top panel) carries a 51 residue glycine-rich linker that resulted from a double insertion of the G5 linker (see Table 1 in the main manuscript).

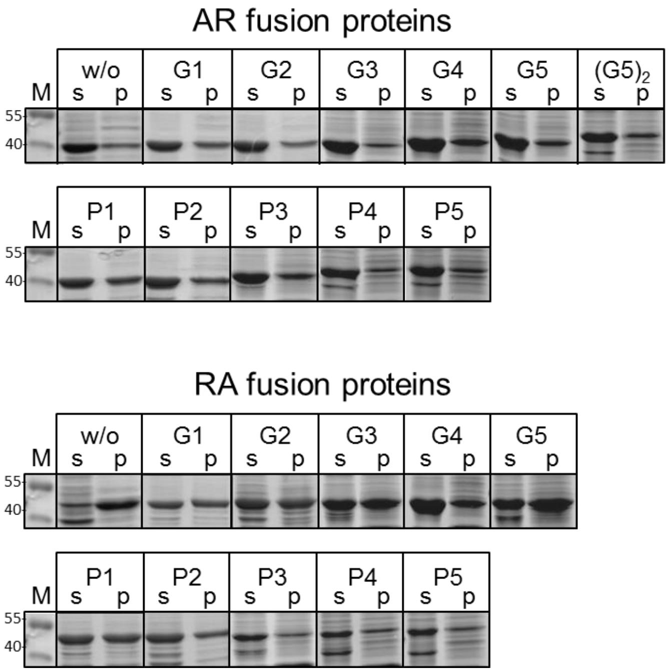

Supplementary Figure S2. ESI-MS analysis of selected fusion constructs. To verify the molecular mass of selected fusion constructs, the purified proteins were dialyzed against ddH_2O for 1 h (2 x 30 min, Scienova Microdialyzer 6 - 8 kDa) and subjected to ESI-MS. ESI-MS was essentially performed as in[2] using a tandem hybrid mass spectrometer (QSTAR XL, Applied Biosystems, Darmstadt, Germany) equipped with an offline nanospray ion source. Mass spectra were typically measured in a mass range 700 – 2500 m/z. The ESI–MS spectra were reconstructed using the deconvolution algorithm included in the analysis software (Applied Biosystems). The theoretical and measured average mass of the various fusion constructs are shown in table below.

Protein	Theoretical average mass (Da)		Measured average mass (Da)
	+Met1	-Met1	
AR	48292.71	48161.52	48631
AR-P4	50462.32	50331.13	50464
RA-P5	51035.03	50903.83	51034

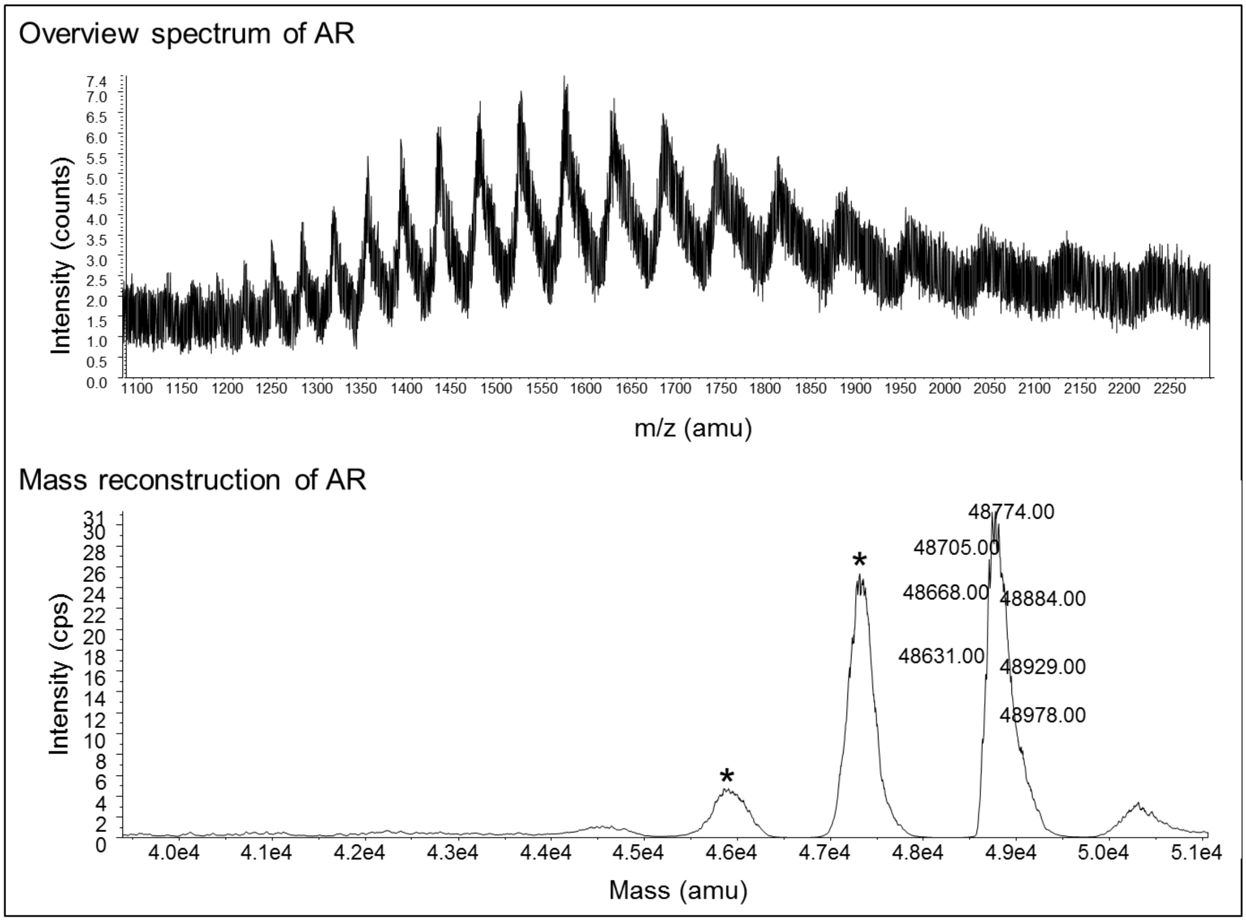

* indicates fusion construct-derived fragments that are formed during ESI-MS measurement.

Overview spectrum of AR-P4

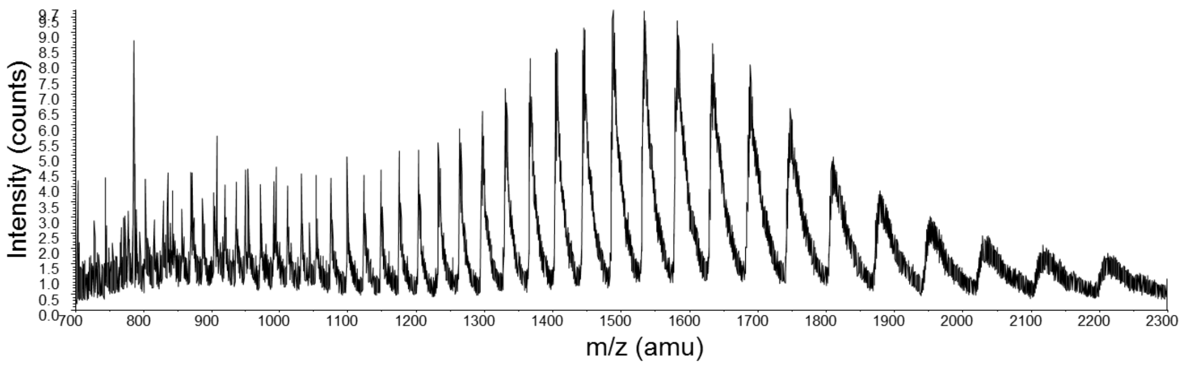

Mass reconstruction of AR-P4

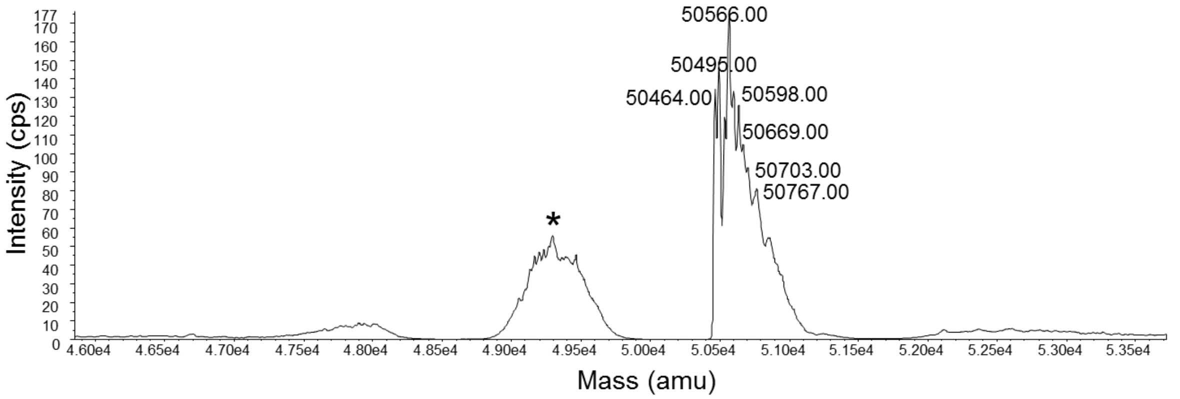

Overview spectrum of RA-P5

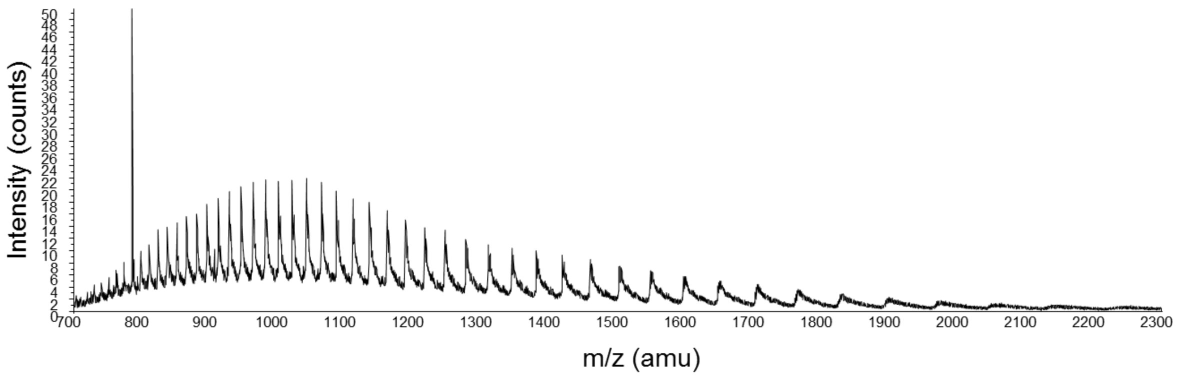

Mass reconstruction of RA-P5

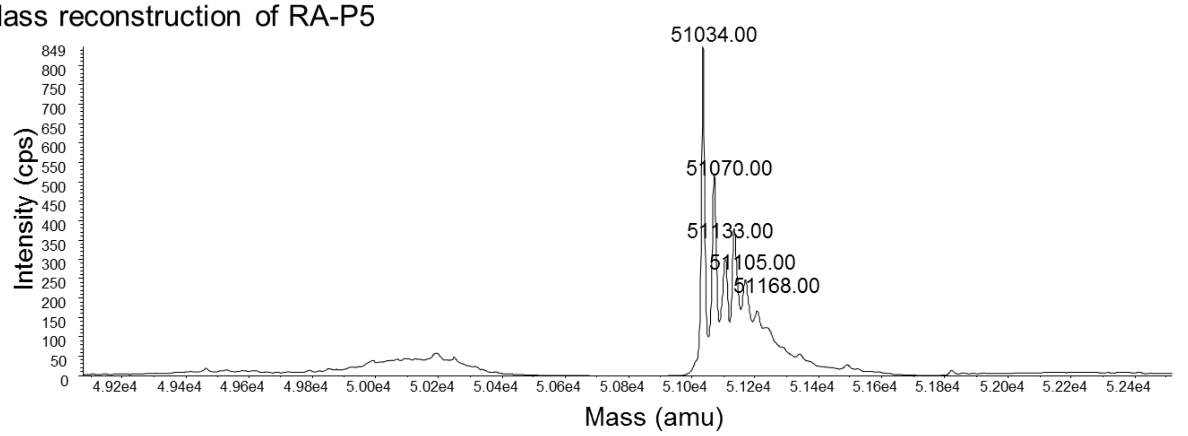

SDS-PAGE analysis of the AR fusion construct indicated an apparent MW of ~40 kDa (Fig. 1, main manuscript), whereas its theoretical mass is 48.293 kDa. ESI-MS analysis of the AR construct revealed however a mass of 48.631 kDa, which demonstrates that truncation of the AR fusion protein had not occurred. A slight mass difference between the theoretical mass and the measured mass was observed, which can be explained by the presence of retained cofactor FMN (456.34 Da) and an additional oxidation (within mass accuracy ± 1.5 Da). The masses of the redox active constructs AR-P4 and RA-P5 determined by ESI-MS were found to be identical to their theoretical masses. Thus ESI-MS analysis confirmed the masses of the fusion constructs. It can therefore be excluded that the MW discrepancy observed for the AR fusion constructs during SDS-PAGE analysis is due to a loss of mass resulting from e.g. protein truncation.

Supplementary Table S1. PCR primers used to fuse the *fldA* and *fpr* genes. Underlined nucleotides indicate restriction sites relevant to cloning, *i.e. Nde*I(CATATG) / *Bam*HI(GGATCC), and linker insertion, *i.e. Nco*I (CCATGG). The overlapping primer pairs 2/3 and 6/7 are fully complementary and lack the stop codon, which allows the in frame fusion of the *fldA* and *fpr* genes, with nucleotides of *fpr* highlighted in grey. To generate an *Nco*I site at the fusion site of *fpr:fldA* three extra nucleotides were incorporated (highlighted in bold). As a result the DNA at the fusion site of both the *fldA:fpr* (AR) and *fpr:fldA* (RA) construct codes for Ala-Met-Ala (see also Fig. 1c of the main manuscript), with minimal disturbance of the natural amino acid sequences.

Primer (name)	Nucleotide sequence
1(fldA-Fw)	5'-*GAGATATACATATG*GCTATCACTGGCATCTTTTTC-3'
2(fldA:fpr-Rv)	5'- GCCTGTTACCCAATCAGCCATGGCATTGAGAATTTCGTCGAGA-3'
3(fldA:fpr-Fw)	5'- TCTCGACGAAATTCTCAATGCCATGGCTGATTGGGTAACAGGC -3'
4(fpr-Rv)	5'-*CAGCCGGATCC*TTACCAGTAATGCTCC-3'
5(fpr-Fw)	5'-*GAGATATACAT*ATGGCTGATTGGGTAACAGGC-3'
6(fpr:fldA-Rv)	5'-AAAGATGCCAGTGATAGCCAT**GGC**CCAGTAATGCTCCGC-3'
7(fpr:fldA-Fw)	5'-GCGGAGCATTACTGG**GCC**ATGGCTATCACTGGCATCTTT-3'
8(fldA-Rv)	5'-*TTAGCAGCCGGATCC*TTAGGCATTGAGAAT-3'
9(h6-fldA-Fw)	5'- *TATACATATG*CATCATCATCATCATCATGCTATCACTGGCATCTTTTTC -3'
10(h6-fpr-Fw)	5'-*CAACAT*ATGCATCATCATCATCATCATGCTGATTGGGTAACAGG-3'

Supplementary Table S2. Plasmids used in this study.

Plasmid	Properties	Reference
pET11a-*fldA*	*E. coli* FldA (no tag)	[1]
pET16b-*fpr*	*E. coli* Fpr with N-terminal His_6-tag	[3]
pET11a-*his:fldA*	*E. coli* FldA with N-terminal His_6-tag, obtained via PCR on pET11a-*fldA* and subsequent cloning via *Nde*I-*Bam*HI	this study
pET11a-*his:fpr*	*E. coli* Fpr with N-terminal His_6-tag, obtained via PCR on pET16b-*fpr* and subsequent cloning via *Nde*I-*Bam*HI	this study
pET11a-*fldA:fpr*	AR fusion construct; obtained by direct fusion of Fpr to the C-terminus of FldA	this study
pET11a-*fpr:fldA*	RA fusion construct; obtained by direct fusion of FldA to the C-terminus of Fpr	this study
pET11a-*hAR*	AR fusion construct carrying N-terminal His_6-tag	this study
pET11a-*hRA*	RA fusion construct carrying N-terminal His_6-tag	this study
pET11a-*hAR-G1*	hAR fusion with linker GGGGS	this study
pET11a-*hAR-G2*	hAR fusion with linker GGGGS-GGGGS	this study
pET11a-*hAR-G3*	hAR fusion with linker GGGGS-GGGGS-GGGGS	this study
pET11a-*hAR-G4*	hAR fusion with linker GGGGS-GGGGS-GGGGS-GGGGS	this study
pET11a-*hAR-G5*	hAR fusion with linker GGGGS-GGGGS-GGGGS-GGGGS-GGGGS	this study
pET11a-*hAR-(G5)$_2$*	hAR fusion with linker GGGGS-GGGGS-GGGGS-GGGGS-GGGGS-M-GGGGS-GGGGS-GGGGS-GGGGS-GGGGS	this study
pET11a-*hRA-G1*	hRA fusion with linker GGGGS	this study
pET11a-*hRA-G2*	hRA fusion with linker GGGGS-GGGGS	this study
pET11a-*hRA-G3*	hRA fusion with linker GGGGS-GGGGS-GGGGS	this study
pET11a-*hRA-G4*	hRA fusion with linker GGGGS-GGGGS-GGGGS-GGGGS	this study
pET11a-*hRA-G5*	hRA fusion with linker GGGGS-GGGGS-GGGGS-GGGGS-GGGGS	this study
pET11a-*hAR-P1*	hAR fusion with linker EPPPP	this study
pET11a-*hAR-P2*	hAR fusion with linker EPPPP-LPPPP	this study
pET11a-*hAR-P3*	hAR fusion with linker EPPPP-EPPPP-LPPPP	this study
pET11a-*hAR-P4*	hAR fusion with linker EPPPP-LPPPP-LPPPP-EPPPP	this study
pET11a-*hAR-P5*	hAR fusion with linker EPPPP-EPPPP-LPPPP-LPPPP-LPPPP	this study
pET11a-*hRA-P1*	hRA fusion with linker EPPPP	this study
pET11a-*hRA-P2*	hRA fusion with linker EPPPP-LPPPP	this study
pET11a-*hRA-P3*	hRA fusion with linker EPPPP-EPPPP-LPPPP	this study
pET11a-*hRA-P4*	hRA fusion with linker EPPPP-LPPPP-LPPPP-EPPPP	this study
pET11a-*hRA-P5*	hRA fusion with linker EPPPP-EPPPP-LPPPP-LPPPP-LPPPP	this study

References

1 Jenkins, C. M. & Waterman, M. R. NADPH-flavodoxin reductase and flavodoxin from *Escherichia coli*: characteristics as a soluble microsomal P450 reductase. *Biochemistry* **37**, 6106-6113 (1998).

2 Horn, C. *et al.* Monitoring conformational changes during the catalytic cycle of OpuAA, the ATPase subunit of the ABC transporter OpuA from Bacillus subtilis. *Biochem. J.* **412**, 233-244, doi:10.1042/BJ20071443 (2008).

3 Girhard, M., Klaus, T., Khatri, Y., Bernhardt, R. & Urlacher, V. B. Characterization of the versatile monooxygenase CYP109B1 from *Bacillus subtilis*. *Appl. Microbiol. Biotechnol.* **87**, 595-607, doi:10.1007/s00253-010-2472-z (2010).
